# Supplementary material for: Empirical methods for controlling false positives and estimating confidence in ChIP-Seq peaks
Source: BMC Bioinformatics. 2008 Dec 5;9:523. doi: 10.1186/1471-2105-9-523 (PMC2628906; doi:10.1186/1471-2105-9-523)
Supplement: Additional File 1 — A variety of html documents from the USeq web site detailing the available applications, their best usage, output file type descriptions, command line menus, etc. [file 1471-2105-9-523-S1.zip › USeqUserGuides/JavaDoc.zip!/JavaDoc/util/bio/parsers/RefSeqAnnotationParser.html]

RefSeqAnnotationParser


|  |  |  |
| --- | --- | --- |
| **Overview** \| **Package** \| **Class** \| **Use** \| **Tree** \| **Deprecated** \| **Index** \| **Help** | |  |
| **PREV CLASS**   **NEXT CLASS** | **FRAMES**    **NO FRAMES**     **All Classes** |  |
| SUMMARY: NESTED \| FIELD \| CONSTR \| METHOD | DETAIL: FIELD \| CONSTR \| METHOD |  |


---


## util.bio.parsers Class RefSeqAnnotationParser

```
java.lang.Object
  util.bio.parsers.RefSeqAnnotationParser
```

---

``` public class RefSeqAnnotationParser extends java.lang.Object ```

Parser for pulling gene annotations into a UCSC table format for Sodalis. Ugly! Better to go with XML!

---

**Constructor Summary**

`RefSeqAnnotationParser()`


| **Method Summary** | |
| --- | --- |
| `java.lang.String` | `find(java.util.HashSet att, java.lang.String prefix)` |
| `static void` | `main(java.lang.String[] args)` |
| `void` | `parseIt(java.io.File file)` |
| `void` | `printUCSCTableFormat(java.util.HashMap hash)` |
| `boolean` | `skipToFeatures(java.io.BufferedReader in)` |
| `java.lang.String` | `splitAndClean(java.lang.String x)` |

**Methods inherited from class java.lang.Object**

`equals, getClass, hashCode, notify, notifyAll, toString, wait, wait, wait`

**Constructor Detail**

### RefSeqAnnotationParser

```
public RefSeqAnnotationParser()
```


**Method Detail**

### printUCSCTableFormat

```
public void printUCSCTableFormat(java.util.HashMap hash)
```

---


### splitAndClean

```
public java.lang.String splitAndClean(java.lang.String x)
```

---


### find

```
public java.lang.String find(java.util.HashSet att,
                             java.lang.String prefix)
```

---


### skipToFeatures

```
public boolean skipToFeatures(java.io.BufferedReader in)
```

---


### parseIt

```
public void parseIt(java.io.File file)
```

---


### main

```
public static void main(java.lang.String[] args)
```


---


|  |  |  |
| --- | --- | --- |
| **Overview** \| **Package** \| **Class** \| **Use** \| **Tree** \| **Deprecated** \| **Index** \| **Help** | |  |
| **PREV CLASS**   **NEXT CLASS** | **FRAMES**    **NO FRAMES**     **All Classes** |  |
| SUMMARY: NESTED \| FIELD \| CONSTR \| METHOD | DETAIL: FIELD \| CONSTR \| METHOD |  |


---
